# Multi-Task Deep Learning Model for Automated Detection and Severity Grading of Lumbar Spinal Stenosis on MRI: Multi-Center External Validation

**DOI:** 10.3390/diseases14010032

**Published:** 2026-01-14

**Authors:** Phatcharapon Udomluck, Watcharaporn Cholamjiak, Jakkaphong Inpun, Waragunt Waratamrongpatai

**Affiliations:** 1 School of Medicine, University of Phayao, Phayao 56000, Thailand; phatcharapon.ud@up.ac.th; 2 School of Science, University of Phayao, Phayao 56000, Thailand; watcharaporn.ch@up.ac.th; 3 School of Information and Communication Technology, University of Phayao, Phayao 56000, Thailand; jakkaphong.564343@gmail.com

**Keywords:** lumbar spinal stenosis, deep feature extraction, machine learning, external validation, MRI imaging

## Abstract

**Background/Objectives**: Accurate and reproducible grading of lumbar spinal stenosis (LSS) is clinically critical for guiding treatment decisions and patient management, yet manual assessment remains challenging due to imaging variability and inter-observer subjectivity. To address these limitations, this study aimed to evaluate the generalizability of deep learning–based feature extraction methods—VGG19, ConvNeXt-Tiny, and DINOv2—combined with classical machine learning classifiers for automated multi-grade LSS assessment. Automated grading enables objective, reproducible, and scalable assessment of lumbar spinal stenosis severity, addressing key limitations of manual interpretation. **Methods**: Axial MRI images were processed using pretrained VGG19, ConvNeXt-Tiny, and DINOv2 models to extract deep features. Logistic Regression, Support Vector Machine (SVM), and LightGBM were trained on internal datasets and externally validated using MRI data from the University of Phayao Hospital. Performance was assessed using accuracy, precision, recall, F1-score, confusion matrices, and multi-class ROC curves. **Results**: VGG19-based features yielded the strongest external performance, with Logistic Regression achieving the highest accuracy (0.9556) and F1-score (0.9558). External validation further demonstrated excellent discrimination, with AUC values ranging from 0.994 to 1.000 across all severity grades. SVM (0.9333 accuracy) and LightGBM (0.9222 accuracy) also performed well. ConvNeXt-Tiny showed stable cross-model performance, while DINOv2 features exhibited reduced generalizability, especially with LightGBM (accuracy 0.6222). Most classification errors occurred between adjacent grades. **Conclusions**: Deep convolutional features—particularly VGG19—combined with classical machine learning classifiers provide robust and generalizable LSS grading across external MRI data. Despite advances in modern architectures, CNN-based feature extraction remains highly effective for spinal imaging and represents a practical pathway for clinical decision support.

## 1. Introduction

Lumbar spinal degenerative disease, particularly lumbar spinal stenosis (LSS), represents one of the most common and debilitating spinal disorders globally, and it is a leading cause of chronic lower back pain, neurogenic claudication, functional decline, and disability in older adults. Its prevalence rises markedly with age—affecting up to 47% of individuals older than 60 years—and LSS contributes substantially to frailty, reduced mobility, and frequent hospitalizations in the aging population [1,2,3,4]. As life expectancy increases worldwide, the clinical and socioeconomic burden of LSS continues to escalate, making accurate detection and severity grading a priority for optimizing both surgical and nonsurgical management strategies [5]. Severity-based stratification is essential because treatment decisions for LSS—including conservative therapy, minimally invasive decompression, or extensive surgical interventions—depend heavily on radiologic grading and anatomical characterization of central canal, foraminal, and lateral recess stenosis [6,7].

Conventional diagnostic workflows rely on radiologists’ interpretation of magnetic resonance imaging (MRI) or computed tomography (CT) scans using qualitative or semi-quantitative grading systems. Although widely adopted, these approaches are limited by inter-reader variability, inconsistent criteria application, and substantial time burden—particularly in busy clinical settings or resource-limited hospitals [8,9,10,11]. With increasing imaging demands and global shortages of specialized radiologists, the need for scalable, automated, and objective diagnostic support tools has become increasingly urgent.

Recent advances in deep learning (DL), particularly convolutional neural networks (CNNs) and transformer-based vision architectures, have fundamentally transformed the landscape of medical image analysis. Classical CNN-based models, including visual geometry group (VGG)-based transfer learning frameworks, have demonstrated strong capability in learning hierarchical and spatially meaningful representations for biomedical imaging tasks [12,13,14,15]. More recently, transformer-based and foundation models, such as Vision Transformers and self-supervised approaches like distillation with no labels, version 2 (DINOv2), have further extended representation learning by capturing global contextual information and robust visual features without explicit supervision [16,17,18,19]. In parallel, recent clinical studies have highlighted the effectiveness of CNN-based and ensemble learning strategies for medical image classification across different disease domains. For example, optimized CNN ensembles and transfer learning approaches have been shown to improve diagnostic accuracy and confidence in mammographic microcalcification detection, demonstrating the clinical reliability of deep feature representations in real-world settings [14]. Similarly, CNN-based feature extraction using pretrained architectures such as VGG and ResNet has been successfully applied to multi-class classification of neurological disorders from structural MRI, emphasizing the general applicability of deep feature learning for disease severity discrimination [20]. Collectively, these advances have led to state-of-the-art performance across a wide range of diagnostic applications, including disease classification, grading, and musculoskeletal MRI interpretation [12,13,14,15,16,17,18,19,20].

Within spinal imaging, DL-based systems have shown promising results in automating disc degeneration grading, vertebral body labeling, spinal canal segmentation, and stenosis quantification [21,22,23,24,25]. However, the majority of existing studies remain constrained by single-center datasets, limited demographic diversity, insufficient multi-class severity stratification, or inadequate consideration of real-world variability in MRI acquisition protocols, scanner hardware, and patient characteristics [26,27]. These limitations raise persistent concerns regarding model generalizability and external applicability, which continue to represent key barriers to reliable clinical translation.

The emergence of large-scale, publicly accessible imaging datasets, such as the Radiological Society of North America (RSNA) 2024 Lumbar Spine Degenerative Classification dataset, provides new opportunities for benchmarking model performance using standardized, expertly annotated MRI images [28]. Public datasets enable reproducibility, reduce barriers to entry for artificial intelligence (AI) development, and support model training without additional ethical concerns because the images are fully anonymized [29]. Nevertheless, exclusive reliance on public data may not capture the heterogeneity of real-world clinical practice, where differences in imaging vendors, acquisition parameters, case-mix severity, and population-specific anatomical variations can substantially affect model performance [30,31,32].

To address these challenges, rigorous external validation using independent datasets is widely recognized as an essential step before clinical deployment of AI systems [33,34,35,36]. Well-designed external validation evaluates model robustness under distribution shifts, ensures reliability across diverse patient populations, and enhances clinical trustworthiness. Studies lacking external validation frequently demonstrate optimistic performance estimates that do not translate effectively into real-world use [37,38,39]. Furthermore, in the context of degenerative spine disease—which is strongly linked to frailty, reduced mobility, falls, and hospitalization among older adults—validated AI tools could play a critical role in early detection, timely intervention, and population-level risk stratification [40,41,42,43,44].

In this study, an advanced multi-task deep learning framework is developed to perform automated detection and severity grading of LSS, integrating multi-class classification of central canal stenosis, lateral recess stenosis, neural foraminal narrowing, and disc degeneration patterns. To ensure clinical relevance and generalizability, the model is first trained on the RSNA 2024 public dataset and subsequently evaluated on an independent external validation cohort comprising 100 anonymized MRI studies from the University of Phayao Hospital. By incorporating real-world, multi-center, heterogeneous imaging data, this research aims to assess not only predictive accuracy but also practical adaptability, reproducibility, and robustness.

Ultimately, the integration of deep learning with methodologically rigorous external validation addresses critical gaps in prior LSS research and supports international recommendations for transparent, reliable, and clinically applicable AI development [40,41]. The findings of this work are expected to contribute to systematic improvements in diagnostic accuracy, radiologist workload reduction, and enhanced decision-making for the management of older adults—particularly those at risk for frailty, mobility impairment, and recurrent hospitalizations.

In contrast to many state-of-the-art studies that primarily focus on end-to-end deep learning architectures evaluated on single-center or internally split datasets, this study emphasizes clinical robustness and external generalizability for automated lumbar spinal stenosis grading. Rather than proposing a new network architecture, we introduce a systematic feature–classifier matrix framework that decouples deep feature extraction from classification, enabling a transparent and comprehensive comparison across multiple representation and decision models. By incorporating independent external validation using real-world MRI data from a different institution, this work provides a more realistic assessment of model stability under heterogeneous imaging conditions. Furthermore, the results demonstrate that combining deep convolutional features with simple, well-regularized, and interpretable classifiers can achieve reliable performance in clinical settings, highlighting an alternative and practically deployable pathway beyond increasingly complex state-of-the-art models.

## 2. Materials and Methods

### 2.1. Study Design and Data Sources

This study employed a multi-stage machine learning pipeline integrating public MRI datasets, expert-reviewed grading criteria, and external validation using real-world imaging data. The primary training dataset was obtained from the RSNA 2024 lumbar spine degenerative classification repository (Kaggle; accessed on 5 September 2025) [28], which contains sagittal lumbar spine MRI images with standardized labels for degenerative severity. According to the RSNA grading system, images were annotated as

1 = Normal/Mild;

2 = Moderate;

4 = Severe.

To ensure compatibility with clinical grading frameworks, we additionally applied an expert-driven severity mapping based on established lumbar nerve root compression criteria.

### 2.2. Expert-Based Severity Grading (Approval Grades A–D)

A spinal imaging specialist independently reviewed and annotated MRI scans using a four-tier categorical system [7,41]:

Grade A (Mild; A1–A4): Nerve roots well separated with preserved cerebrospinal fluid (CSF) signal.

Grade B (Moderate): Nerve roots demonstrating early clustering; cerebrospinal fluid (CSF) space partially reduced.

Grade C (Severe): Nerve roots compressed, with CSF signal absent; epidural fat may be present.

Grade D (Extreme): Severe compression with indistinguishable nerve roots; absence of both CSF and epidural fat.

This mapping was utilized to enrich the RSNA labels and align them with clinically interpretable severity categories.

### 2.3. Image Preprocessing and Quality Control

All images underwent preprocessing before model development:

#### 2.3.1. Structural Similarity Index (SSIM) Filtering

To ensure consistent image quality and remove noisy or corrupted scans, SSIM [45] was applied as a quality control metric. Images below a predefined SSIM threshold were excluded from training to reduce the risk of model misclassification.

#### 2.3.2. Data Augmentation [46]

To address class imbalance and enhance generalization, augmented training samples were generated using controlled transformations, including

Rotation (±10°);

Horizontal/vertical shifts;

Random cropping;

Contrast-limited adaptive histogram equalization (CLAHE).

Augmentation was applied uniformly across severity classes to mitigate overfitting [43].

### 2.4. Feature Extraction Using Deep Learning Models

Following preprocessing, deep feature representations were extracted from three state-of-the-art deep learning architectures:

VGG19 [15]—a classical convolutional neural network pretrained on ImageNet;

ConvNeXt-Tiny [47]—a modern, efficient convolutional backbone with improved hierarchical representations;

DINOv2 [19]—a self-supervised vision transformer (ViT) approach enabling robust feature extraction under limited labels.

Each model was used as a fixed feature extractor, generating high-dimensional embeddings subsequently utilized for downstream machine learning classification.

The selection of these architectures was guided by their complementary representation paradigms. VGG19 represents a classical CNN architecture that captures hierarchical texture and morphological features, which are particularly relevant to anatomical pattern recognition in spinal MRI. ConvNeXt-Tiny embodies recent advances in convolutional design, offering improved inductive biases and enhanced feature abstraction while retaining spatial locality. In contrast, DINOv2 was included to assess the suitability of transformer-based, self-supervised representations for domain-specific medical imaging tasks, enabling a comparative analysis between convolutional and transformer-driven feature learning strategies. Multiple feature extraction methods were intentionally employed to capture complementary visual representations and to systematically evaluate the robustness of automated lumbar spinal stenosis grading across different deep learning paradigms.

### 2.5. Machine Learning Classification

Extracted features were used to train three complementary machine learning models:

Logistic Regression (LR) [48]—baseline linear classifier;

Support Vector Machine (SVM) [49]—using radial basis kernel;

LightGBM [50]—gradient boosting framework optimal for tabular, high-dimensional feature sets.

Model hyperparameters were optimized via 5-fold cross-validation on the training set. Performance metrics included accuracy, precision, recall, F1-score, and AUC where applicable.

These classifiers were selected to represent distinct decision-making mechanisms with varying levels of complexity and interpretability. Logistic Regression was chosen for its linear formulation, robustness, and interpretability, making it well suited for clinically oriented decision-support systems. SVM provides margin-based non-linear classification, allowing for flexible separation in complex feature spaces. LightGBM, as a tree-based ensemble method, was included to model non-linear feature interactions and assess the effectiveness of boosting-based approaches when combined with deep feature representations. Together, this combination enables a systematic evaluation of how different feature extractors interact with diverse classification paradigms under a unified experimental framework.

### 2.6. External Validation Using MRI from University of Phayao Hospital

To evaluate real-world generalizability, the final models were externally validated using 70 anonymized MRI lumbar spine images collected from the University of Phayao Hospital. MRI examinations were performed using a 1.5-Tesla superconducting magnet system (MAGNETOM Aera; Siemens Healthcare, Erlangen, Germany). The scanner was equipped with a 24-channel spine matrix coil and a gradient system with a maximum amplitude of 57 mT/m and a slew rate of 216 T/m/ms. All images were fully de-identified according to data privacy regulations and approved for secondary analysis. The external dataset included:

Sagittal T1- and T2-weighted MRI sequences;

Ground truth severity classification by a board-certified radiologist independent of the research team;

External validation was conducted without additional fine-tuning to assess model robustness under domain shift conditions.

### 2.7. Workflow Overview

The proposed methodology is organized as a systematic multi-stage pipeline that integrates data curation, quality control, imbalance handling, deep feature extraction, and machine learning–based classification, followed by independent external validation.

**Figure 1:**
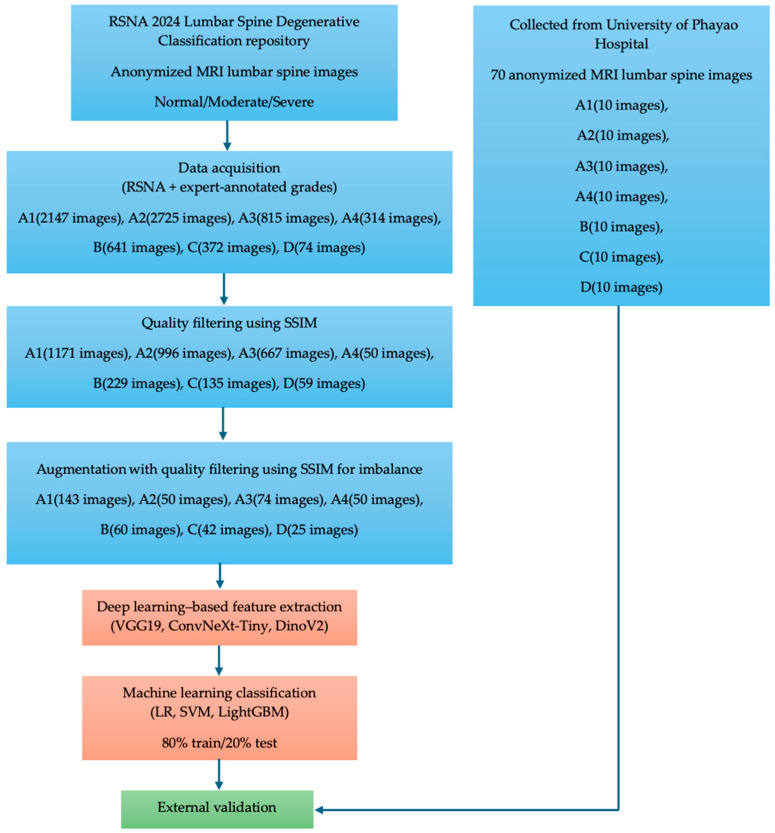
Workflow of the multi-stage deep learning and machine learning pipeline for automated detection and severity grading of lumbar spinal stenosis.

From Figure 1, lumbar spine MRI data were collected from two sources. The primary dataset was obtained from the RSNA 2024 Lumbar Spine Degenerative Classification repository, which provided anonymized lumbar spine MRI images for model development. All images were annotated according to expert-defined severity grades following the RSNA criteria and mapped into a seven-class grading scheme (A1–A4, B, C, and D). The initial class distribution before quality control consisted of A1 (2147 images), A2 (2725 images), A3 (815 images), A4 (314 images), B (641 images), C (372 images), and D (74 images). To ensure data consistency and reliability, a quality filtering step based on the Structural Similarity Index Measure (SSIM) was applied. Images with low structural similarity or inconsistent quality were excluded, resulting in a curated dataset with the following class distribution: A1 (1171 images), A2 (996 images), A3 (667 images), A4 (50 images), B (229 images), C (135 images), and D (59 images). This step explicitly defines the exclusion criteria used during dataset construction. Subsequently, data augmentation with additional SSIM-based quality control was applied to mitigate class imbalance. Augmentation was selectively performed to balance the seven severity classes rather than to uniformly increase the dataset size. After this imbalance-handling stage, the final dataset used for model training and testing consisted of A1 (143 images), A2 (50 images), A3 (74 images), A4 (50 images), B (60 images), C (42 images), and D (25 images). Deep feature representations were then extracted using three complementary architectures—VGG19, ConvNeXt-Tiny, and DINOv2—representing classical convolutional, modern convolutional, and transformer-based design paradigms, respectively. The extracted feature embeddings were subsequently classified using three machine learning classifiers: logistic regression (LR), support vector machines (SVM), and LightGBM. For model evaluation, the dataset was split into 80% training and 20% testing, and classification performance was assessed using confusion matrices reflecting the seven-class grading scheme. Finally, an independent external validation was conducted using 70 anonymized lumbar spine MRI images collected from the University of Phayao Hospital, with 10 images per class (A1–A4, B, C, and D), to evaluate real-world generalizability.

This structured combination enabled a comprehensive comparison across all feature–classifier pairs, facilitating a clear analysis of their relative performance and generalization ability. Finally, the best-performing configurations were evaluated using an external clinical dataset to assess real-world applicability and robustness.

All experiments were conducted with fixed implementation settings to ensure reproducibility. Image quality filtering was performed using SSIM with a threshold of 0.80, below which images were excluded. Data augmentation included random rotations (±10°), horizontal flipping (probability = 0.5), and brightness/contrast adjustments within ±15%. Feature extraction was performed using a batch size of 16. Hyperparameters for each classifier were optimized via 5-fold cross-validation, and the final settings are reported in the manuscript. Experiments were executed on a workstation equipped with an NVIDIA 3050 labtop 4 gb (NVIDIA Corporation, Santa Clara, CA, USA), CPU AMD Ryzen 5 5600H processor with integrated Radeon Graphics (Advanced Micro Devices, Inc., Santa Clara, CA, USA), and 16 GB of system RAM.

## 3. Results

To evaluate the effect of different deep feature extraction strategies on model performance, we compared three pretrained architectures—VGG19, ConvNeXt-Tiny, and DINOv2—as fixed feature extractors. Each model produced high-dimensional embeddings that were subsequently classified using Logistic Regression, Support Vector Machine (SVM), and LightGBM. The objective was to determine how traditional CNN-based features (VGG19), modern hierarchical convolutional representations (ConvNeXt-Tiny), and self-supervised transformer embeddings (DINOv2) influenced downstream classification performance.

This subsection presents the results obtained using VGG19-based features, followed by results from ConvNeXt-Tiny and DINOv2 in the subsequent tables. Performance metrics include accuracy, precision, recall, and F1-score for both training and test sets.

### 3.1. Performance of Machine Learning Classifiers Using Deep Feature Extraction from Multiple Pretrained Models

Table 1 presents a unified comparison of all deep feature extraction and classifier combinations, providing a clear overview of both training performance and generalization behavior. Across all feature extractors, near-perfect training performance was consistently observed, indicating that the extracted deep embeddings were highly discriminative. Differences among classifiers became evident on the test set. For VGG19-based features, LightGBM achieved the strongest generalization performance (accuracy = 0.8989, F1-score = 0.8968), while Logistic Regression showed moderate robustness and SVM suffered from pronounced performance degradation, suggesting overfitting.

In contrast, ConvNeXt-Tiny embeddings yielded more stable generalization across classifiers. Both Logistic Regression and SVM achieved high and comparable test accuracy (0.9326) with F1-scores exceeding 0.93, indicating improved robustness to unseen data, whereas LightGBM exhibited slightly lower but still competitive performance. For DINOv2-based features, Logistic Regression consistently demonstrated the most stable generalization (accuracy = 0.9326, F1-score = 0.9327), followed by SVM, while LightGBM showed reduced performance relative to the other feature extractors.

Overall, the combined results highlight that generalization performance is jointly influenced by both the choice of deep feature extractor and the classifier. While all models achieved excellent training accuracy, Logistic Regression emerged as the most consistently stable classifier across different feature representations, whereas LightGBM performance was more feature-dependent.

**Figure 2:**
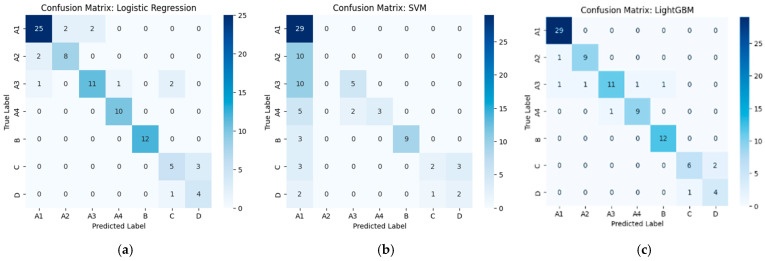
Confusion matrices of machine learning classifiers using VGG19-based deep feature extraction. (**a**) Logistic Regression shows generally accurate predictions with minor confusion between adjacent grades. (**b**) SVM exhibits substantial misclassification across multiple classes. (**c**) LightGBM provides the most accurate and stable predictions with minimal class overlap.

From Figure 2, using VGG19-based features, Logistic Regression achieves reasonable classification performance with limited confusion between neighboring grades, whereas SVM suffers from pronounced misclassification and class bias. LightGBM consistently outperforms the other models, yielding the clearest diagonal structure and minimal class overlap, particularly for higher severity grades.

**Figure 3:**
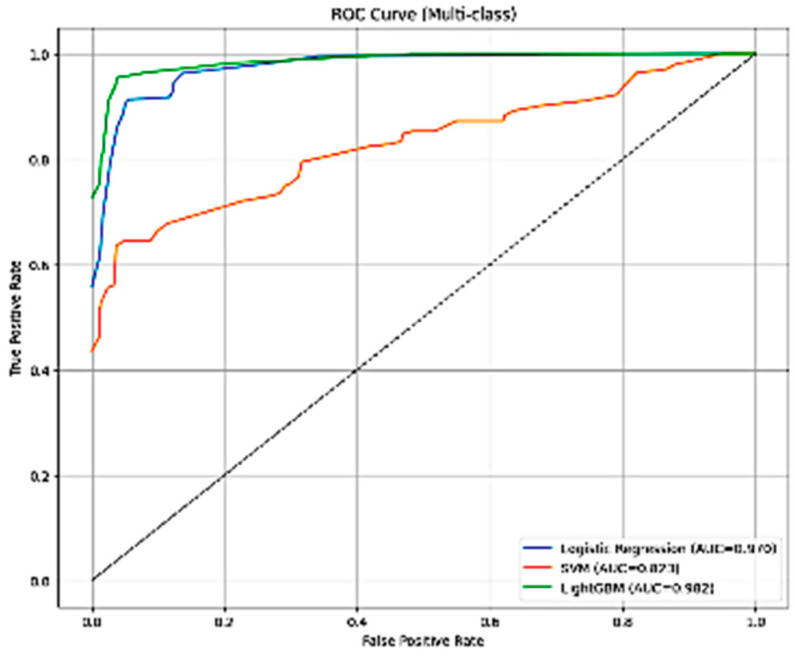
Multi-class ROC curves for Logistic Regression, SVM, and LightGBM using VGG19-based deep feature extraction.

From Figure 3, the multi-class ROC analysis demonstrates clear differences in classifier performance. Logistic Regression and LightGBM both produced high AUC values, indicating strong discriminative ability across the A1–D severity grades. LightGBM achieved the highest AUC (0.9021), followed by Logistic Regression (0.9100), while SVM showed substantially lower performance (AUC = 0.8721). LightGBM also exhibited the most stable and smooth ROC trajectory near the upper-left region, reflecting robust generalization. In contrast, SVM showed a markedly lower ROC curve, consistent with its poorer test-set performance and higher misclassification rate. These findings confirm that gradient boosting (LightGBM) is the most effective classifier when combined with VGG19-derived deep features.

**Figure 4:**
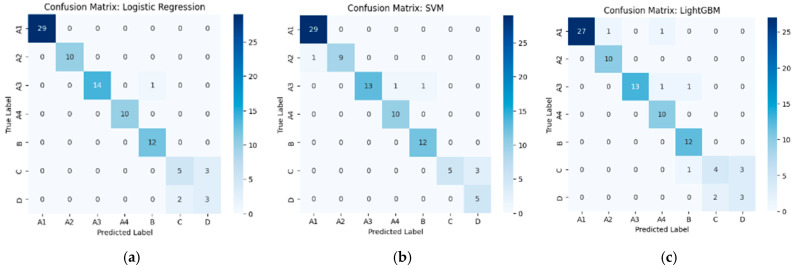
Confusion matrices of ML classifiers using ConvNeXt-Tiny features. (**a**) Logistic Regression, (**b**) SVM, and (**c**) LightGBM.

From Figure 4, confusion matrices derived from ConvNeXt-Tiny deep features show that all three classifiers—Logistic Regression, SVM, and LightGBM—achieve strong performance with clear diagonal dominance, indicating reliable separation of lumbar stenosis severity grades (A1–D). Logistic Regression and SVM provide slightly cleaner boundaries among the A1–A4 mild subgrades, whereas LightGBM demonstrates minor confusion between classes C and D. Overall, ConvNeXt-Tiny–based features support consistently high classification accuracy across models.

**Figure 5:**
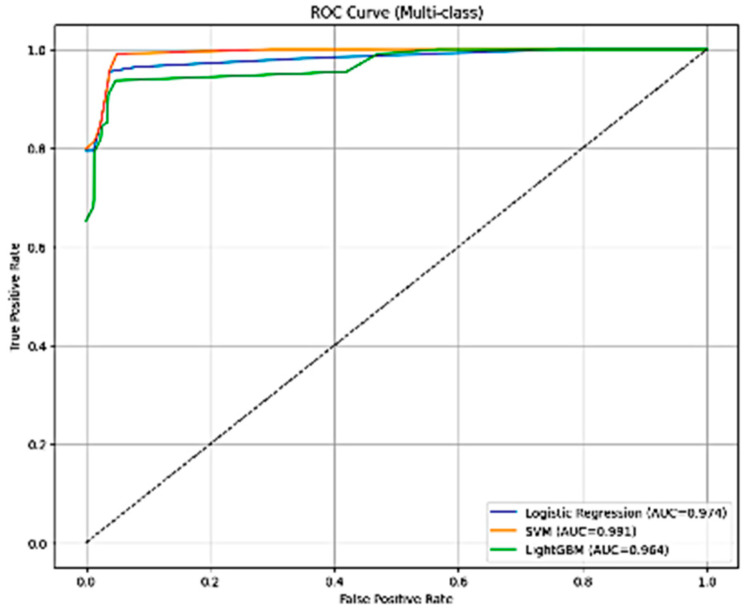
Multi-class ROC curves for classifiers using ConvNeXt-Tiny deep features.

From Figure 5, the ROC analysis indicates that all three classifiers achieve strong multi-class discrimination when using ConvNeXt-Tiny–derived features. SVM achieves the highest AUC (0.981), followed closely by Logistic Regression (0.974), while LightGBM shows slightly lower performance (AUC = 0.964). The steep rise and near-ceiling trajectories of all curves reflect excellent sensitivity across severity classes, confirming the robustness of ConvNeXt-based feature extraction for automated stenosis grading.

**Figure 6:**
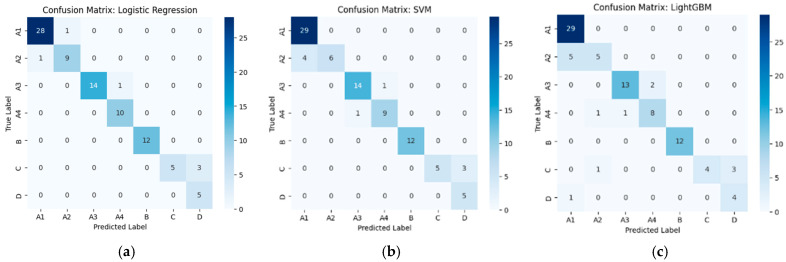
Confusion matrices of ML classifiers using DinoV2 features. (**a**) Logistic Regression, (**b**) SVM, and (**c**) LightGBM.

From Figure 6, the confusion matrices derived from DinoV2-based deep feature extraction show that all three classifiers—Logistic Regression, SVM, and LightGBM—successfully distinguish the A1–D severity grades with strong diagonal dominance. Logistic Regression provides the cleanest separation across classes, particularly for the A1–A4 mild categories and grade B. SVM demonstrates comparable performance, though with slightly increased confusion between A3–A4 and C–D. LightGBM exhibits the highest degree of misclassification, especially in the C and D categories, aligning with its lower test-set metrics. Overall, DinoV2 features support robust classifier performance, with Logistic Regression emerging as the most stable and accurate model.

**Figure 7:**
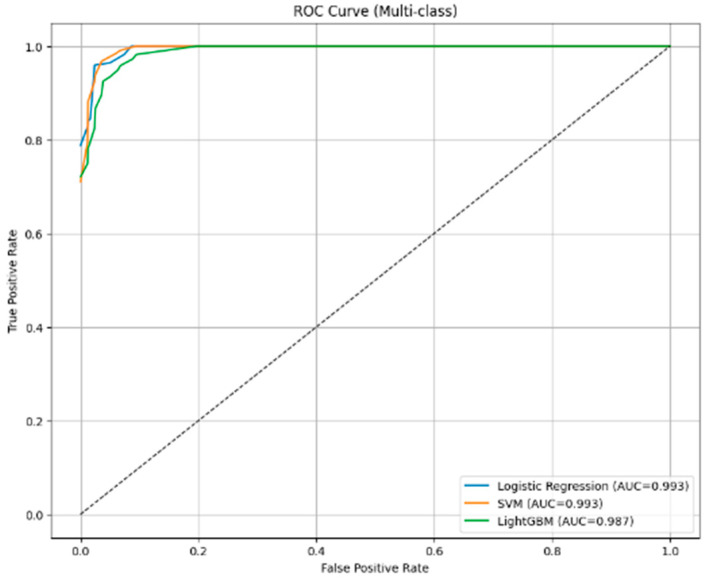
Multi-class ROC curves for classifiers using DinoV2 deep features.

From Figure 7, the multi-class ROC curves indicate that all classifiers achieve excellent discriminative performance when using DinoV2-based deep features. Logistic Regression and SVM yield nearly identical AUC values (AUC = 0.993), reflecting highly accurate separation across all severity grades (A1–D). LightGBM also performs strongly (AUC = 0.987), though with a slightly lower curve in the early false-positive region, indicating marginally reduced sensitivity compared to the other two models. Overall, DinoV2 features provide highly robust representations, enabling consistently high classification performance across all machine learning algorithms.

### 3.2. External Validation Using MRI Data from University of Phayao Hospital

To evaluate real-world generalizability, all models trained on the RSNA dataset were externally validated using 70 anonymized MRI lumbar spine images collected from the University of Phayao Hospital. This dataset, annotated according to severity grades A1–D by experienced radiologists, reflects heterogeneous imaging protocols and patient characteristics encountered in clinical practice. The following section reports model performance on this independent cohort.

#### 3.2.1. Performance of Machine Learning Classifiers Using VGG19-Based Deep Features

**Figure 8:**
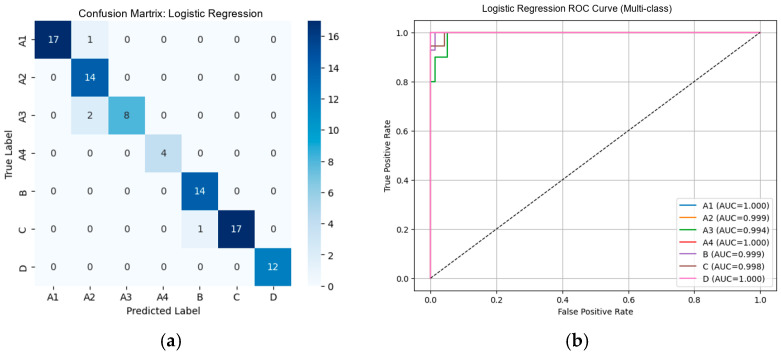
External validation of Logistic Regression using VGG19 features: (**a**) Confusion matrix and (**b**) multi-class ROC curves showing near-perfect discrimination (AUC = 0.994–1.000).

**Figure 9:**
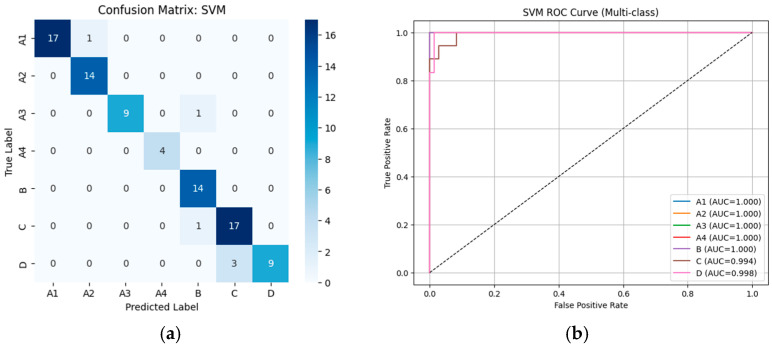
External validation of SVM using VGG19 features: (**a**) Confusion matrix and (**b**) multi-class ROC curves showing near-perfect discrimination (AUC = 0.994–1.000).

**Figure 10:**
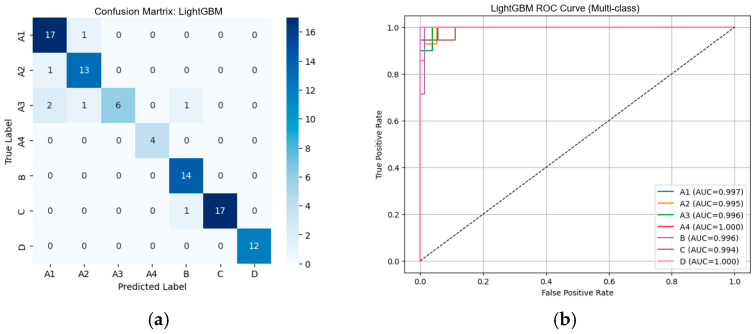
External validation of LightGBM using VGG19 features: (**a**) Confusion matrix and (**b**) multi-class ROC curves showing near-perfect discrimination (AUC = 0.994–1.000).

From Table 2, the external validation using MRI images from the University of Phayao Hospital demonstrates strong generalizability of all three classifiers, despite variations in patient characteristics and imaging protocols. Logistic Regression achieved the highest overall performance, with Accuracy = 0.9556, Precision = 0.9622, Recall = 0.9556, and F1-score = 0.9558, indicating excellent stability when combined with VGG19 deep features. SVM showed slightly lower performance (Accuracy = 0.9333; F1-score = 0.9326) but remained highly competitive, reflecting reliable discrimination across severity grades. LightGBM, while still performing well (Accuracy = 0.9222; F1-score = 0.9190), exhibited the lowest metrics among the three models, suggesting reduced robustness on heterogeneous real-world data. Overall, these findings indicate that VGG19-based feature extraction paired with Logistic Regression provides the most accurate and reliable framework for external clinical deployment in automated grading of lumbar spinal stenosis. Figure 8, Figure 9 and Figure 10 present the confusion matrices and corresponding multi-class ROC curves obtained using Logistic Regression, SVM, and LightGBM, respectively.

#### 3.2.2. Performance of Machine Learning Classifiers Using Convnext Tiny-Based Deep Features

**Figure 11:**
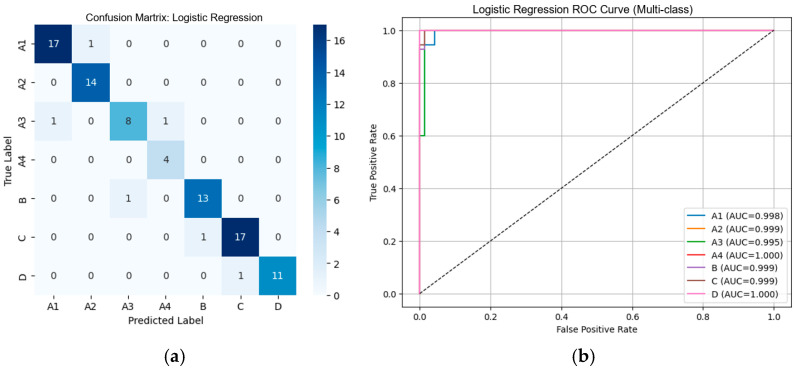
External validation of Logistic Regression using ConvNeXt-Tiny features: (**a**) Confusion matrix and (**b**) multi-class ROC curves showing near-perfect discrimination (AUC = 0.995–1.000).

**Figure 12:**
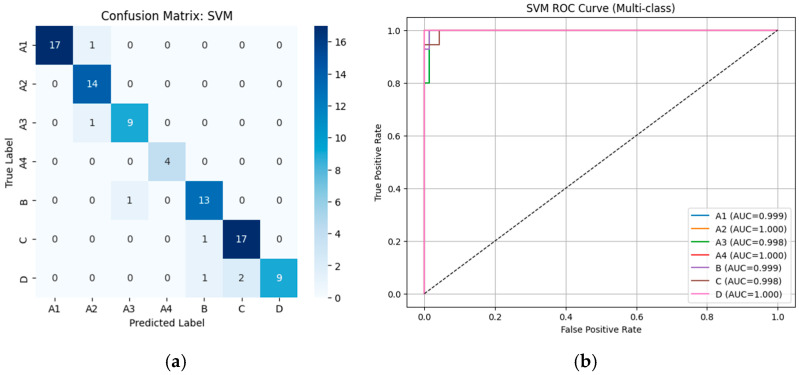
External validation of SVM using ConvNeXt-Tiny features: (**a**) Confusion matrix and (**b**) multi-class ROC curves showing near-perfect discrimination (AUC = 0.998–1.000).

**Figure 13:**
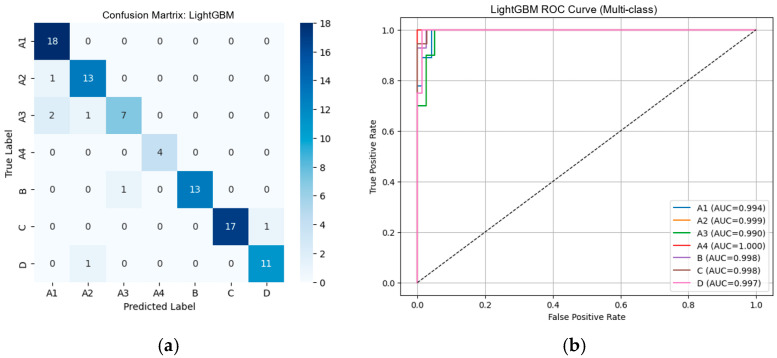
External validation of LightGBM using ConvNeXt-Tiny features: (**a**) Confusion matrix and (**b**) multi-class ROC curves showing near-perfect discrimination (AUC = 0.994–1.000).

From Table 3, external validation using the ConvNeXt-Tiny deep features demonstrated strong and consistent performance across all classifiers. Logistic Regression achieved the highest overall performance (Accuracy = 0.9333, F1-score = 0.9330), indicating effective generalization to real-world MRI data from the University of Phayao Hospital. Both SVM (Accuracy = 0.9222, F1-score = 0.9214) and LightGBM (Accuracy = 0.9222, F1-score = 0.9212) also performed well, though slightly lower than Logistic Regression in all metrics. Figure 11, Figure 12 and Figure 13 present the confusion matrices and corresponding multi-class ROC curves obtained using Logistic Regression, SVM, and LightGBM, respectively.

#### 3.2.3. Performance of Machine Learning Classifiers Using Dinov2-Based Deep Features

**Figure 14:**
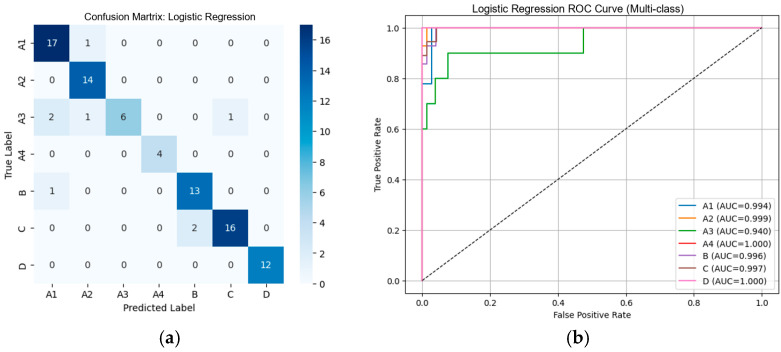
External validation of Logistic Regression using DinoV2 features: (**a**) Confusion matrix and (**b**) multi-class ROC curves showing near-perfect discrimination (AUC = 0.940–1.000).

**Figure 15:**
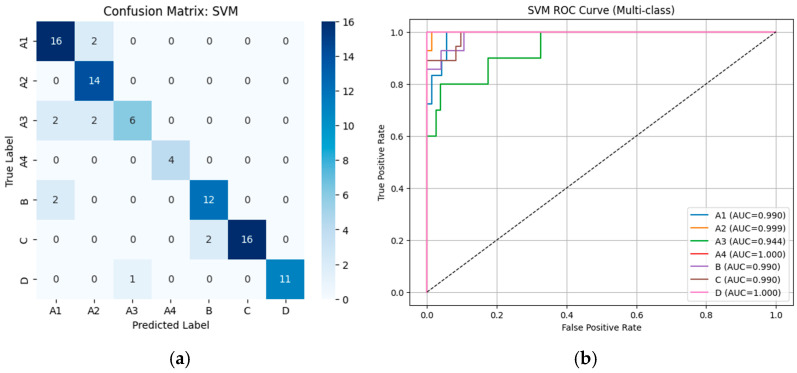
External validation of SVM using DinoV2 features: (**a**) Confusion matrix and (**b**) multi-class ROC curves showing near-perfect discrimination (AUC = 0.944–1.000).

**Figure 16:**
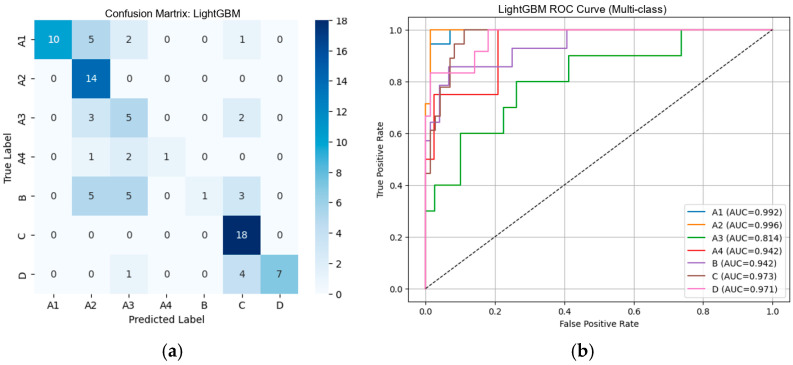
External validation of SVM using DinoV2 features: (**a**) Confusion matrix and (**b**) multi-class ROC curves showing near-perfect discrimination (AUC = 0.942–1.000).

From Table 4, external validation with DinoV2-based deep features showed a clear performance hierarchy among the three classifiers. Logistic Regression achieved the best overall results (Accuracy = 0.9111, F1-score = 0.9076), demonstrating strong generalization to real-world MRI data. SVM exhibited moderately high performance (Accuracy = 0.8778, F1-score = 0.8765), though consistently lower than Logistic Regression across all metrics. In contrast, LightGBM showed substantial performance degradation (Accuracy = 0.6222, F1-score = 0.5843), indicating poor alignment between DinoV2 feature representations and tree-based learning in the external testing scenario. Overall, DinoV2 features supported strong performance for linear and kernel-based models, with Logistic Regression emerging as the most robust and reliable classifier for external clinical deployment. Figure 14, Figure 15 and Figure 16 present the confusion matrices and corresponding multi-class ROC curves obtained using Logistic Regression, SVM, and LightGBM, respectively.

### 3.3. Representative Examples from VGG19-Based Deep Feature Predictions

**Figure 17:**
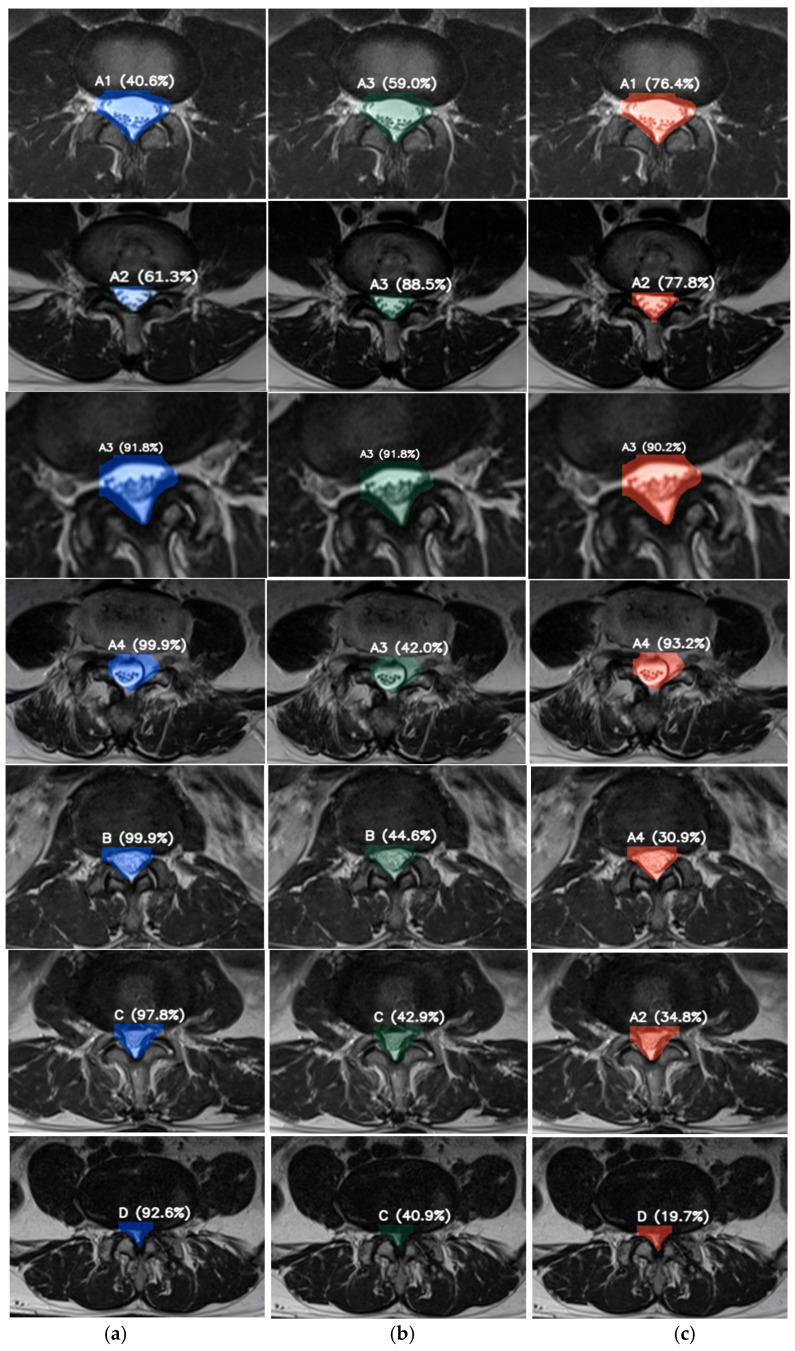
Example predictions using VGG19-based deep features across three machine learning classifiers: (**a**) Logistic Regression, (**b**) SVM, and (**c**) LightGBM. The predicted stenosis grade and associated confidence score are shown on each MRI slice.

Figure 17 illustrates representative axial lumbar spine MRI slices illustrating the inference-stage classification results using VGG19-based deep feature extraction combined with three machine learning classifiers: (a) Logistic Regression, (b) SVM, and (c) LightGBM. For each slice, the predicted lumbar spinal stenosis severity grade (A1–D) and the corresponding confidence score are displayed. The highlighted regions indicate the central spinal canal area, which provides the key anatomical information used by the model for feature extraction and classification.

These visual examples demonstrate how the proposed framework operates during real-world inference, translating deep feature representations into clinically interpretable severity grades. Correct predictions with high confidence reflect robust feature–classifier alignment, whereas lower-confidence or misclassified cases typically occur between adjacent severity grades, illustrating the inherent difficulty of borderline stenosis assessment. Overall, the figure provides qualitative evidence of the model’s decision behavior and supports the clinical relevance and interpretability of the proposed automated grading system.

## 4. Discussion

This study presents a systematic evaluation of three deep feature extraction strategies—VGG19, ConvNeXt-Tiny, and DINOv2—combined with classical machine learning classifiers for automated severity grading of lumbar spinal stenosis, with particular emphasis on external validation using independent clinical MRI data from the University of Phayao Hospital. By organizing the analysis within a unified feature–classifier matrix, the findings provide insight into how representation learning and classifier choice jointly influence generalization in real-world clinical settings.

Across all experiments, near-perfect training performance was consistently observed, confirming the strong discriminative capacity of the extracted deep features. However, meaningful differences emerged during external validation, underscoring the importance of evaluating models beyond internal performance metrics. In this context, VGG19- and ConvNeXt-Tiny–based features demonstrated superior robustness, while DINOv2-based representations showed greater variability depending on the downstream classifier. The strong and stable performance of VGG19-derived features across classifiers—particularly when paired with Logistic Regression—suggests that convolutional features learned from structured spatial hierarchies remain highly effective for spinal MRI analysis. Despite being a comparatively older architecture, VGG19 appears well aligned with the anatomical and morphological characteristics relevant to lumbar spinal stenosis, such as canal narrowing, ligament hypertrophy, and disc-related compression patterns. This stability across institutions highlights its suitability as a dependable backbone for clinical imaging pipelines, where data heterogeneity is unavoidable. ConvNeXt-Tiny features also demonstrated robust generalization, especially when combined with simpler linear or margin-based classifiers. This indicates that modern convolutional architectures can offer improved representational power while maintaining compatibility with interpretable and computationally efficient classifiers. In contrast, DINOv2—although highly effective in self-supervised natural image benchmarks—showed reduced generalization in this task, particularly with tree-based boosting methods. This suggests that transformer-style embeddings may not optimally capture the subtle, domain-specific anatomical variations required for stenosis severity grading without further task-specific adaptation.

Across all feature representations, Logistic Regression consistently exhibited the most stable generalization behavior. Its robustness may be attributed to its linear decision boundaries, which reduce sensitivity to overfitting and distributional shifts commonly encountered in multi-center clinical data. From a clinical deployment perspective, this finding is particularly relevant, as Logistic Regression offers transparency, ease of calibration, and predictable behavior—key requirements for clinical decision-support systems. In contrast, SVM showed moderate sensitivity to feature distribution changes, while LightGBM demonstrated stronger dependence on the nature of the extracted features, performing well with convolution-derived embeddings but less reliably with transformer-based representations. From a clinical standpoint, the observed misclassification patterns further support the practical relevance of the proposed framework. Errors were predominantly confined to adjacent severity grades (e.g., A3 vs. A4 or C vs. D), which mirrors real-world diagnostic challenges where borderline cases exhibit overlapping morphological characteristics. This suggests that the proposed feature-based ML approach effectively captures core anatomical cues associated with stenosis severity, while remaining challenged by subtle transitional cases that often require expert radiological judgment.

Recent state-of-the-art approaches increasingly rely on deep convolutional and transformer-based or end-to-end learning architectures and have demonstrated strong performance on internal benchmarks in medical image analysis. Classical CNNs, such as U-Net and VGG, established the effectiveness of hierarchical feature learning for biomedical and visual recognition tasks [14,15], while more recent transformer-based models, including Vision Transformers and self-supervised foundation models such as DINOv2, have shown powerful representation learning capabilities [16,19]. Nevertheless, despite their success on curated benchmarks, these models often require extensive fine-tuning and large, relatively homogeneous datasets, which may limit robustness under heterogeneous real-world clinical imaging conditions. In this context, the inclusion of ConvNeXt-Tiny and DINOv2 in our study provides representative comparisons with modern convolutional and transformer-based paradigms, while emphasizing generalizability under external validation rather than peak performance.

The superior external validation performance of VGG19 can be attributed to its convolutional architecture, which emphasizes local texture and edge-based features that are highly relevant to spinal MRI interpretation. Lumbar spinal stenosis grading relies on subtle morphological changes, such as canal narrowing, disc protrusion, and ligament hypertrophy, which are effectively captured by hierarchical convolutional filters. In contrast, although DINOv2 demonstrates strong performance in natural-image benchmarks, its transformer-based global representations may be less sensitive to fine-grained anatomical variations in grayscale medical imaging without domain-specific adaptation. Furthermore, the observed misclassification patterns, predominantly occurring between adjacent severity grades, reflect the inherent clinical ambiguity of borderline stenosis cases. Such errors align with known inter-observer variability among radiologists and suggest that the proposed model captures core anatomical cues while remaining challenged by transitional disease stages. This reinforces the potential role of the system as a clinical decision-support tool rather than a replacement for expert judgment.

The consistently stable generalization observed with Logistic Regression can be attributed to its linear decision boundaries and inherent regularization, which reduce sensitivity to noise and distributional shifts in high-dimensional deep feature spaces. When combined with discriminative deep embeddings, Logistic Regression effectively leverages the representational power of deep networks while avoiding excessive model complexity. In contrast, more flexible classifiers such as SVM and LightGBM may be more sensitive to feature distribution changes or prone to overfitting, particularly under limited or heterogeneous clinical data. From a clinical deployment perspective, the robustness, interpretability, and predictable behavior of Logistic Regression further support its suitability for real-world decision-support systems.

While end-to-end fine-tuning of pre-trained models has demonstrated strong performance on internally curated datasets, prior studies have reported that such approaches may be prone to overfitting and reduced robustness when applied to heterogeneous clinical data and cross-institutional settings [14,15,16,19]. In this study, our objective was not to maximize internal accuracy, but to evaluate generalizability under external validation. By decoupling feature extraction from classification, the proposed framework reduces model complexity and variance, thereby supporting more stable performance across institutions, which is particularly important for real-world clinical deployment.

Overall, these findings suggest that integrating deep feature extraction with simple and interpretable classifiers offers a clinically robust alternative to increasingly complex architectures. This strategy achieves a balanced trade-off between performance, generalizability, and interpretability, which is critical for real-world clinical deployment. As such, feature-based machine learning pipelines show strong potential for supporting automated lumbar spinal stenosis grading as assistive tools in routine clinical workflows, including screening, triage, and the reduction in inter-observer variability.

## 5. Conclusions

This study systematically evaluated three deep feature extraction approaches—VGG19, ConvNeXt-Tiny, and DINOv2—in combination with classical machine learning classifiers for automated severity grading of lumbar spinal stenosis. By adopting a structured feature–classifier matrix and emphasizing external validation, the study provides a realistic assessment of model generalizability in clinical imaging environments.

Across all experiments, convolution-based feature extractors demonstrated superior robustness compared with transformer-based representations. In particular, VGG19-derived features consistently achieved strong external validation performance, while ConvNeXt-Tiny also exhibited stable and competitive generalization. In contrast, DINOv2 features showed reduced performance across classifiers, suggesting limited suitability for capturing the subtle, domain-specific anatomical patterns required for spinal stenosis grading without additional task-specific adaptation. Among classifiers, Logistic Regression emerged as the most stable and reliable model across different feature representations. Its consistent performance highlights the advantage of combining expressive deep features with simple, well-regularized classifiers when deploying decision-support systems in heterogeneous, real-world clinical settings. The observed misclassification patterns, predominantly occurring between adjacent severity grades, further reflect the inherent diagnostic ambiguity of borderline stenosis cases and align with known clinical challenges.

Overall, the findings indicate that feature-based machine learning pipelines remain a practical, interpretable, and robust strategy for automated lumbar spinal stenosis grading, particularly when external generalizability is a critical requirement. Future research should focus on larger multi-center datasets, domain adaptation strategies to improve cross-institutional robustness, and hybrid or end-to-end deep learning frameworks incorporating attention mechanisms to further enhance classification reliability and clinical interpretability.

## Figures and Tables

**Table 1:** Performance comparison of machine learning classifiers across different deep feature extraction methods for automated grading of lumbar spinal stenosis.

Feature Extractor	Classifier	Train Acc	Train Prec	Train Rec	Train F1	Test Acc	Test Prec	Test Rec	Test F1
VGG19	Logistic Regression	1.0000	1.0000	1.0000	1.0000	0.8427	0.8487	0.8427	0.8436
	SVM	1.0000	1.0000	1.0000	1.0000	0.5618	0.6024	0.5618	0.5069
	LightGBM	1.0000	1.0000	1.0000	1.0000	0.8989	0.9005	0.8989	0.8968
ConvNeXt-Tiny	Logistic Regression	1.0000	1.0000	1.0000	1.0000	0.9326	0.9359	0.9326	0.9333
	SVM	0.9915	0.9927	0.9915	0.9916	0.9326	0.9475	0.9326	0.9321
	LightGBM	1.0000	1.0000	1.0000	1.0000	0.8876	0.8937	0.8876	0.8863
DINOv2	Logistic Regression	1.0000	1.0000	1.0000	1.0000	0.9326	0.9462	0.9326	0.9327
	SVM	0.9831	0.9851	0.9831	0.9833	0.8989	0.9170	0.8989	0.8947
	LightGBM	1.0000	1.0000	1.0000	1.0000	0.8427	0.8535	0.8427	0.8346

**Table 2:** Performance of machine learning classifiers using VGG19-based deep features on external MRI data from University of Phayao Hospital.

Model	Accuracy	Precision	Recall	F1-Score
Logistic Regression	0.9556	0.9622	0.9556	0.9558
SVM	0.9333	0.9402	0.9333	0.9326
LightGBM	0.9222	0.9298	0.9222	0.9190

**Table 3:** Performance of machine learning classifiers using ConvNeXt-Tiny–based deep features on external MRI data from University of Phayao Hospital.

Model	Accuracy	Precision	Recall	F1-Score
Logistic Regression	0.9333	0.9351	0.9333	0.9330
SVM	0.9222	0.9277	0.9222	0.9214
LightGBM	0.9222	0.9257	0.9222	0.9212

**Table 4:** Performance of machine learning classifiers using DinoV2-based deep features on external MRI data from University of Phayao Hospital.

Model	Accuracy	Precision	Recall	F1-Score
Logistic Regression	0.9111	0.9181	0.9111	0.9076
SVM	0.8778	0.8873	0.8778	0.8765
LightGBM	0.6222	0.7767	0.6222	0.5843

## Data Availability

The RSNA 2024 Lumbar Spine Degenerative Classification dataset is publicly available at https://www.kaggle.com/competitions/rsna-2024-lumbar-spine-degenerative-classification/overview (accessed on 5 September 2025). The retrospective hospital dataset from the University of Phayao Hospital is not publicly available due to ethical restrictions but can be obtained upon reasonable request to the corresponding author with appropriate ethics approval.
